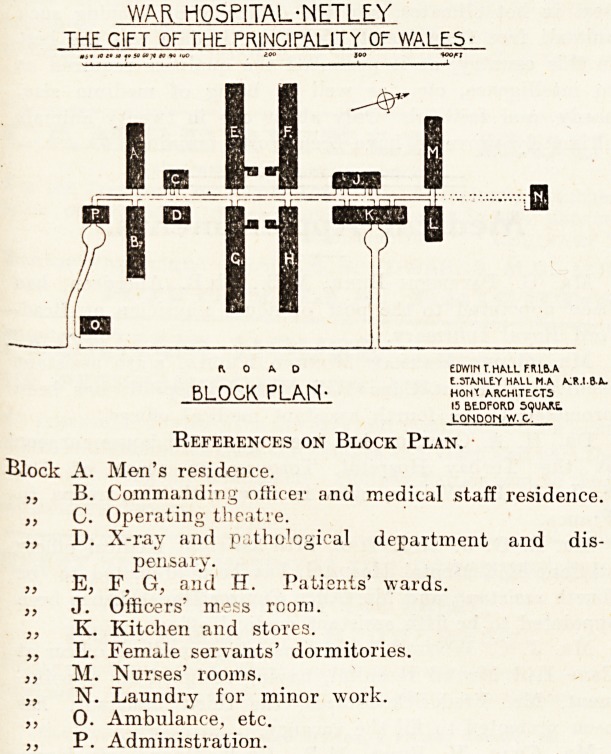# The Welsh War Hospital, Netley: The Movable Hospital to Be Opened Next Week

**Published:** 1914-10-24

**Authors:** 


### THE WELSH WAR HOSPITAL, NETLEY.

### The Movable Hospital to be Opened Next Week.

This is a temporary hospital and has been specially designed so that it can, if necessary, be removed to France or elsewhere. The plan of each building and the relation of the buildings one to another have been settled with the commanding officer, who is a distinguished surgeon. The mortuary block is at the entrance, the pathological laboratory being placed off the main corridor opposite the operation theatre block but across the corridor, and is situated between the block devoted to the quarters of the medical staff and the main ward pavilions. This is not a permanent hospital, and, it is explained, "many arrangements have had to be made to ensure speedy erection, a minimum of parts and other things." The architects were only given instructions about a fortnight before

References on Block Plan.

Block A. Men's residence.
,, B. Commanding officer and medical staff residence.
,, C. Operating theatre.
,, D. X-ray and pathological department and dispensary.
,, E, F, G, and H. Patients' wards.
,, J. Officers' mess room.
,, K. Kitchen and stores.
,, L. Female servants' dormitories.
,, M. Nurses' rooms.
,, N. Laundry for minor work.
,, O. Ambulance, etc.
,, P. Administration.

the hospital was started, and it is estimated that the time occupied between the commencement of the construction to the date of readiness for occupation will not have been more than six weeks. The hospital is expected to begin work at Netley on October 26. Drainage, gas and water mains, all the plumbing, fittings, cooking apparatus, heating and hot-water supply have had to be designed and completed in this short space of time, which has added immensely to the labour of the architects, Messrs. E. T. and E. S. Hall, who are working in an honorary capacity. Credit is due in the case of this hospital, as in that of other similar hospitals, for the record time in which the architects have accomplished most difficult and special work of a novel character.

We are indebted to the architects for the following details:—The Welsh Hospital, Netley, is the gift of the Principality of Wales to H.R.H. the Prince. Its four wards provide accommodation for 104 patients, and provision is made for adding four other wards if necessary.

The buildings are disposed on both sides of a central covered way. They are of a temporary character, so that they may be moved if necessary to another place, and are constructed externally of iron with felted roofs, and internally of asbestos sheets. The contractors are Messrs. Humphreys, of Knightsbridge. The plumbing and sanitary fittings are of course as complete as for a permanent hospital. The buildings will be lighted by electricity throughout, and heated generally by gas radiators. The hot-water supplies throughout are by califonts. The drainage is connected to the general drainage of Netley Hospital.

The site is to the north-east of the Netley Hospital property. The total cost of the hospital w[illegible] be between £6,500 and £7,000, exclusive of furniture and fittings.

### APPOINTMENTS TO THE STAFF.

Lieut.-Colonel A. W. Sheen, M.S., F.R.C.S., R.A.M.C.T., has been appointed Commanding Officer and Senior Surgeon. He is well known in Wales as a consulting surgeon, and is surgeon to King Edward VII.'s Hospital, Cardiff, and consulting surgeon to several other hospitals. He served in the Boer War as surgeon to the Imperial Yeomanry Field Hospital, and until recently commanded the 2nd Welsh Field Ambulance. He is also County Director of Voluntary Aid in Glamorgan. Colonel Sheen is giving honorary service so far as the funds of the Welsh Hospital are concerned.

The medical officers are Mr. Fergus Armstrong, M.B., Ch.B. Edin., F.R.C.S. Edin., of Treorchy, formerly resident medical officer at King Edward VII.'s Hospital, Cardiff, and member of the Edinburgh University Officers Training Corps; Mr. T. Garfield Evans, M.D., B.S. Lond., of Port Talbot, formerly house surgeon, Guy's Hospital, London; Mr. Bernard G. Klein, M.A., M.D., B.Ch. Oxon., of London, assistant pathologist, St. Bartholomew's Hospital and Seamen's Hospital, Greenwich, and formerly house physician, St. Bartholomew's Hospital; Mr. J. Sydney Rowlands, M.D. Liverpool, of Cardiff, anæsthetist to King Edward VII.'s Hospital, Cardiff, formerly house surgeon, house physician, and resident medical officer at the Liverpool Royal Infirmary, and lieutenant in the 2nd Welsh Field Ambulance.

The quartermaster is Mr. E. J. August, of London. Mr. August, who has had eighteen years' service with the 1st Volunteer Battalion Surrey Rifles, was until recently manager of the National Provincial Bank, Cardiff.

The medical officers and quartermaster are to be given commissions in the R.A.M.C.

The dressers are Messrs. L. W. Jones, Brynsiencyn, Anglesey; T. D. Morgan, Cardiff; and A. A. Prichard, son of Dr. R. Prichard, Cardiff.

The matron is Miss M. Martin, of London. She was trained at the Middlesex Hospital, and was at one time a sister at the Cardiff Infirmary. Miss Martin served in the Boer War as a nursing sister in the Welsh Hospital, and is now the head of a large nursing home in London.

The dispenser is Miss Olive E. Williams, King Edward VII.'s Hospital, Cardiff.

## Figures and Tables

**Figure f1:**